# How to measure and record blood pressure

**Published:** 2013

**Authors:** Dianne Pickering, Sue Stevens

**Affiliations:** Nurse Advisor (retired), *Community Eye Health Journal*dianne_logan@hotmail.com; Nurse Advisor (retired), *Community Eye Health Journal*

**Figure:**
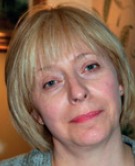
Dianne Pickering

**Figure:**
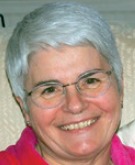
Sue Stevens

This article will explain how to measure and record blood pressure using a **sphygmomanometer** (Figure 1). There are many other types of machines for recording blood pressure, such as electronic devices, but these may not be readily available. They can also be difficult to maintain and therefore may give inaccurate readings.

## What is blood pressure?

Blood pressure is the force of blood against the walls of the arteries. Blood pressure is recorded as two numbers, the **systolic** pressure (the pressure when the heart beats) over the diastolic pressure (the pressure when the heart relaxes between beats).

We record this with the **systolic pressure first (on the top)** and the **diastolic pressure second (below)**. For example, if the systolic pressure is 120 mmHg (millimetres of mercury) and the diastolic pressure is 80 mmHg, we would describe the blood pressure as ‘120 over 80’, written 120/80.

All patients must be assessed for fitness before they undergo surgery. As part of this assessment, it important to measure and record the patient's blood pressure. There are two reasons for this:

1. It provides an initial recording (a ‘baseline’). If the blood pressure falls suddenly below this baseline after surgery, we are alerted to the fact that the patient may be experiencing complications.
2. It allows us to confirm that the patient is fit enough to undergo surgery. A high blood pressure reading, or indeed a very low blood pressure reading, could suggest that the patient has other medical problems, e.g. an undiagnosed heart condition. He or she may need further medical tests and possibly medication to stabilise the blood pressure before undergoing surgery.

When measuring a patient's blood pressure, the nurse should be aware of factors that can affect the reading and possibly give a false reading, which could lead to unnecessary medical investigations. These factors include:

- blood pressure cuff is too small or is placed over clothing
- the patient has recently exercised
- the patient is cold or otherwise uncomfortable (e.g., they may need to use the toilet first)
- the patient has consumed alcohol or caffeine less than 30 minutes before the reading
- the patient is anxious or stressed
- the patient is talking during the procedure.

**Figure:**
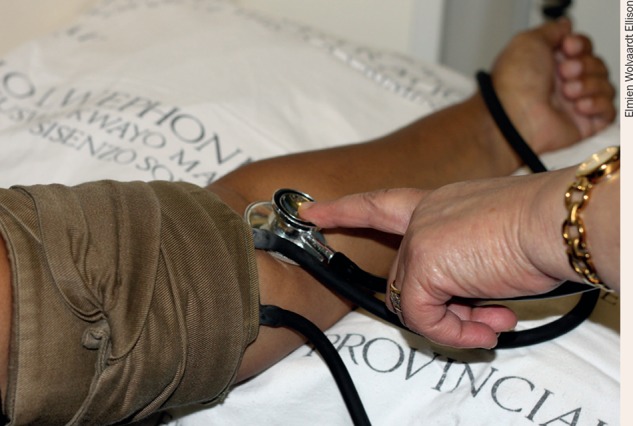
Figure 1. Sphygmomanometer (wall-mounted)

**Figure:**
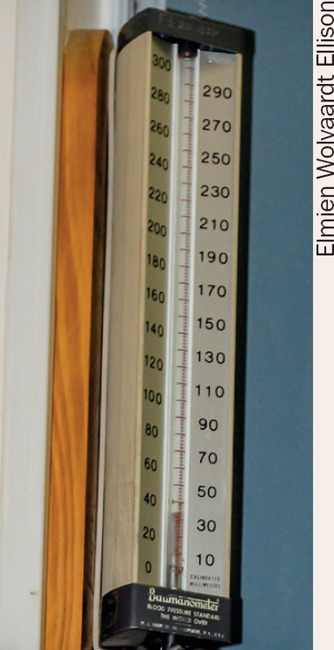
Figure 2. The arm is supported on a level surface. The cuff is around the upper arm and the stethoscope is over the brachial artery, in the bend of the elbow

Blood pressure may vary according to whether the patient is lying down, sitting or standing. It is normally recorded with the patient sitting.

## You will need

- sphygmo-manometer
- blood pressure cuffs: small, medium, large
- stethoscope
- chair
- patient's care notes or observation chart
- alcohol wipe

## Preparation

- Ask whether the patient needs the toilet.
- Ask the patient to sit down. The patient should have rested for 3–5 minutes before starting the procedure.
- Wash and dry your hands.
- Explain to the patient what you are going to do. This will help reduce their anxiety.
- Explain the sensation of the cuff tightening on their arm and reassure them that this is safe.

## Method

- Ask the patient to loosen any tight clothing or remove long-sleeved garments so that it is possible to access the upper arm. Do not use an arm that may have a medical problem.
- Place the cuff around the upper arm and secure.
- Connect the cuff tubing to the sphygmo-manometer tubing and secure.
- Rest the patient's arm on a surface that is level with their arm.
- Place the stethoscope over the brachial artery (in the bend of the elbow) and listen to the pulse (Figure 2).
- Pump up the cuff slowly and listen for when the pulse disappears. This is an indication to stop inflating the cuff.
- Start to deflate the cuff very slowly whilst watching the mercury level in the sphygmomanometer.
- Note the sphygmomanometer reading (the number the mercury has reached) when the pulse reappears: record this as the systolic pressure.
- Deflate the cuff further until the pulse disappears: record this reading as the diastolic pressure.
- Record these two measurements, first the systolic and then the diastolic (e.g., 120/80), in the patient's notes or chart.
- Tell the patient the blood pressure reading.
- Disinfect the stethoscope drum and ear pieces with the alcohol wipe.
- Wash and dry your hands.
- Report an extremely low or high reading to the clinically qualified person in charge of the patient's care.

